# Change in number of pain sites - which factors are important? A 12-year prospective cohort study

**DOI:** 10.1186/s12891-024-07344-x

**Published:** 2024-03-18

**Authors:** Susanne Vilsbøl, David Høyrup Christiansen, Cecilie Rud Budtz, Johan Hviid Andersen, Søren Mose

**Affiliations:** 1 School of Physiotherapy, VIA University College, Holstebro, Denmark; 2 Centre for Research in Health and Nursing, Research, Regional Hospital Central Jutland, Viborg, Denmark; 3 Department of Clinical Medicine, Health, Aarhus University, Aarhus, Denmark; 4 Elective Surgery Center, Silkeborg Regional Hospital, Silkeborg, Denmark; 5 Department of Occupational Medicine, University Research Clinic, Danish Ramazzini Center, Goedstrup Hospital, Aarhus University, Herning, Denmark

**Keywords:** Musculoskeletal pain, Number of pain sites, Chronic pain, Cohort study

## Abstract

**Background:**

Pain in multiple body sites is common and often persistent. The purpose of this prospective study was to examine the change in the number of pain sites (NPS) over time and to evaluate to which extent clinical, demographic, lifestyle and health-related factors predict a change in NPS.

**Methods:**

This was a population-based longitudinal cohort study of adults (*n* = 2,357). Data on pain, demographic, lifestyle, and health-related variables were collected by questionnaires in 2008 and 2020 and register data from 2006 to 2017. Data was analysed with linear regression.

**Results:**

We found a mean decrease in NPS over the 12-year follow-up period (-0.36 (95% CI; -0.44; -0.27) and 56% of this sample reported no change or only one pain site increase/decrease over 12 years. While participants reporting pain for less than 3 months at baseline had almost no change in NPS (-0.04 (95% CI; -0.18; 0.10)), participants with pain for longer than 3 months decreased by -0.51 (95% CI; -0.62; -0.41). Age at baseline (20–49 years), pain intensity, and obesity (BMI ≥ 30) were associated with an increase in NPS over the follow-up period.

**Conclusions:**

NPS is relatively stable over time. We found a small mean decrease in NPS over 12 years varying between participants with pain for longer than 3 months and pain for less than 3 months respectively. The results also indicate that pain intensity, age, and obesity could be relevant factors to consider when predicting change in NPS.

**Supplementary Information:**

The online version contains supplementary material available at 10.1186/s12891-024-07344-x.

## Background

Musculoskeletal (MSK) pain is a prevalent and common complaint in populations worldwide [1–4]. MSK pain is often classified based on location (e.g., low back pain or shoulder pain), even though localized MSK pain is relatively rare and MSK pain often coexists in other body regions [5–10]. This is important because the sum of pain complaints has been shown to be more important than the location of pain in determining the functional and health-related consequences of pain [11–14]. Moreover, the location of pain seems to add little predictive value to more general prognostic factors for poor outcomes across pain locations [15]. MSK pain in multiple body sites is thought to have a persisting pattern over time [6, 16–31] and relatively few with MSK pain in multiple sites fully recover [6, 18, 21, 24]. Nevertheless, a significant number of people tend to experience that number of pain sites (NPS) either increase [16, 19–21, 25, 31] or decrease [6, 18, 21, 22, 25, 29–31] over time, which may indicate that change in NPS might depend on other factors and hence be modifiable. Some studies have investigated potential predictors of change in NPS [16, 18, 24, 25]. As an example is pain intensity associated with a change in NPS over time [6, 19, 28]. Among Norwegian employees higher intensity of headache was associated with an increase in NPS over time while lower pain intensity was associated with a decrease [19]. Sex, age, educational level, psychological disorders, lifestyle factors (obesity, physical activity, smoking), comorbidity, and poor health have also been found to be prognostic factors for higher NPS [6, 16, 21, 23, 32]. However, these findings are inconsistent and sometimes contradictory. Furthermore, it is not clear how the duration of pain influences the persistence of NPS over time. The aim of this study is therefore to (1) describe how the number of MSK pain sites change over a 12-year period in a population-based Danish cohort, (2) explore if such change differs by duration of pain and (3) explore to which extent clinical, demographic, lifestyle, and health-related factors predict the change in NPS. Specifically, we hypothesized that people with a duration of pain for longer than three months would experience less change in the number of pain sites when compared to those with a duration of pain for less than three months.

## Methods

### Design

This is a population-based longitudinal cohort study with 12 years of follow-up. The Strengthening the Reporting of Observational Studies in Epidemiology (STROBE) statement was used as a guide for the reporting of this study [33].

### Study population

Participants in this study are from a population-based representative cohort of working-age Danes. The cohort was established in February 2008. Participants were recruited from the same medical center in the city of Odder, Denmark via a web or postal mailed questionnaire. The questionnaire was sent out to 8,517 individuals between 17 and 64 years of age and 5,068 (59%) individuals responded. Detailed descriptions of the cohort and baseline responders/non-responders have been published elsewhere [7, 34, 35]. In brief, the proportion of women and the mean age were higher among baseline responders than non-responders [34]. The baseline questionnaire collected information on socio-demographics, health (including questions about pain), lifestyle, and psychosocial factors. In October 2020, a web-based follow-up questionnaire was mailed to all 4,765 alive and domestic living baseline responders using the Danish secure digital mailbox system called E-boks. In October 2020, 93% of the population in Odder, Denmark were signed up for E-boks [36]. The follow-up questionnaire collected information about health and work-related factors.

### Outcome

Pain was measured using the Danish version of the Standard Evaluation Questionnaire (SEQ), which is a reliable and valid tool for pain assessment in observational population-based studies [37, 38]. The first section was used to measure the number of pain sites in seven different body regions (head, left/right upper and lower extremity, front/back thorax). The participants were asked to state pain intensity on a seven-point Likert scale (1 = no pain, 7 = worst imaginable pain) during the last four weeks for each body region. NPS was generated by counting body regions with pain regardless of intensity. NPS were then summed, ranging from 0 to 7 at baseline and 0–7 at follow-up and the change in number of pain sites was estimated by the difference between NPS in 2020 minus NPS in 2008 (ranging from − 7 to + 7). Change in NPS was analyzed as a continuous variable.

### Independent baseline variables

All the independent variables were measured in 2008. The selection of independent baseline variables for this study was based on the literature [6, 16, 19–22, 25, 27, 28, 30–32].

Duration of pain at baseline was measured within SEQ dichotomized with a cut-off at 3 months (Pain for less than 3 months/Pain for longer than 3 months) [39].

Pain intensity was measured within SEQ [37]. The highest reported pain intensity in any region was extracted (range 2–7) and pain intensity was analyzed as a continuous variable.

Body Mass Index (BMI) was calculated according to self-reported data on height and weight. Three categories of BMI were used i) “Under/Normal weight (< 25 kg/m^2^), ii) “overweight (25-<30 kg/m^2^)”, iii)” Obesity ≥ 30 kg/m^2^)”.

Comorbidity was obtained by applying the register-based Charlson comorbidity index [40] to ICD10 diagnostic codes from the Danish National Patient Register (NPR) [41]. The comorbidity index was dichotomized into (i) “0 - no comorbidity” or (ii) “1 - comorbidity”. The baseline index was calculated using the previous 2 years of NPR data.

Risk of anxiety and depression was measured by The Common Mental Disorder Questionnaire (CMDQ) [42]. CMDQ is a validated case-finding instrument for common mental disorders useable in primary care settings. The subscale SCL-ANX4 was used for risk of anxiety and subscale SCL-DEP6 was used for risk of depression [42]. The subscale questions were rated on a five-point Likert scale ranging from “not at all” to “extremely”. Both variables were dichotomized into low or high risk, according to recommendations by the Danish College of General Practitioners [43].

Self-reported general health was measured using the subscale for General Health perception (GH) from the 12-item Short-Form Health Survey (SF-12) [44]. GH was used as a raw score and was transformed into a 0 to 100 scale [45]. A higher score indicates better general health [32, 44]. GH was analyzed as a continuous variable.

### Demographic variables

Sex and age at baseline were obtained from the Danish Civil Personal Registration (CPR) System [41]. All persons in Denmark are assigned a unique CPR number, which contains information on sex and age. Age was analysed in six categories (< 20, 20–29, 30–39, 40–49, 50–59, ≥ 60 years).

Level of education achieved was obtained from The Danish Education Register [46]. The International Standard Classification of Education [47] was used for classifying the level of education into three ordinal groups: (i) Bachelor/Master/Doctoral or equivalent, (ii) Upper secondary education or skilled worker/short-cycle tertiary education or equivalent, and (iii) Primary and lower secondary education or equivalent.

### Statistical analysis

Descriptive statistics of the population for all independent variables were presented as frequencies and percentages for binary and categorical variables and mean with standard deviation (SD) or medians with interquartile ranges (IQR). Differences between participants and non-participants (Responders/Non-responders and Participants/Excluded due to missing values) were tested using Pearson’s chi^2^ for binary or categorical variables and Wilcoxon Signed rank test for numerical variables described by medians.

Change in the number of pain sites from baseline (2008) to follow-up (2020) was described by means with 95% confidence interval (95% CI) for all participants and stratified by duration of pain (Pain for less than 3 months/Pain for longer than 3 months). The distribution of change in NPS is presented by graph charts.

For aims 2 and 3, we used linear regression analysis to determine baseline factors predicting change in NPS over 12 years, including determining whether change in NPS was associated with duration of pain. All estimates are presented as regression coefficients with 95% confidence interval (95% CI). Linear regression was done in two models: Model I - Crude analysis with association between each independent variable and outcome and Model II - adjusted analysis included all the independent variables.

As a sensitivity analysis, we compared estimates from the primary full case regression analysis with results from a regression analysis on multiple imputation data. For this analysis, we performed chained multiple imputation techniques imputing ten datasets. Chained multiple imputation was applied on 1,058 (32%) cases missing values on self-reported variables. 22% of these cases had missing values on one variable, 62% on two variables, and 16% on three or more variables. Most missing values were on the change in NPS (74%, 785 cases). Only results from the full case regression analysis are presented.

Assumptions for all regression analyses were controlled visually using diagnostic plots which indicated that the assumptions were met. Significance levels were set to 5%.

We also performed adjusted regression analysis with GH and pain intensity as categorical variables to explore the consequences of data management. All analyses were performed using Stata v.16.1.

## Results

### Non-responders and missing values

**Fig. 1:**
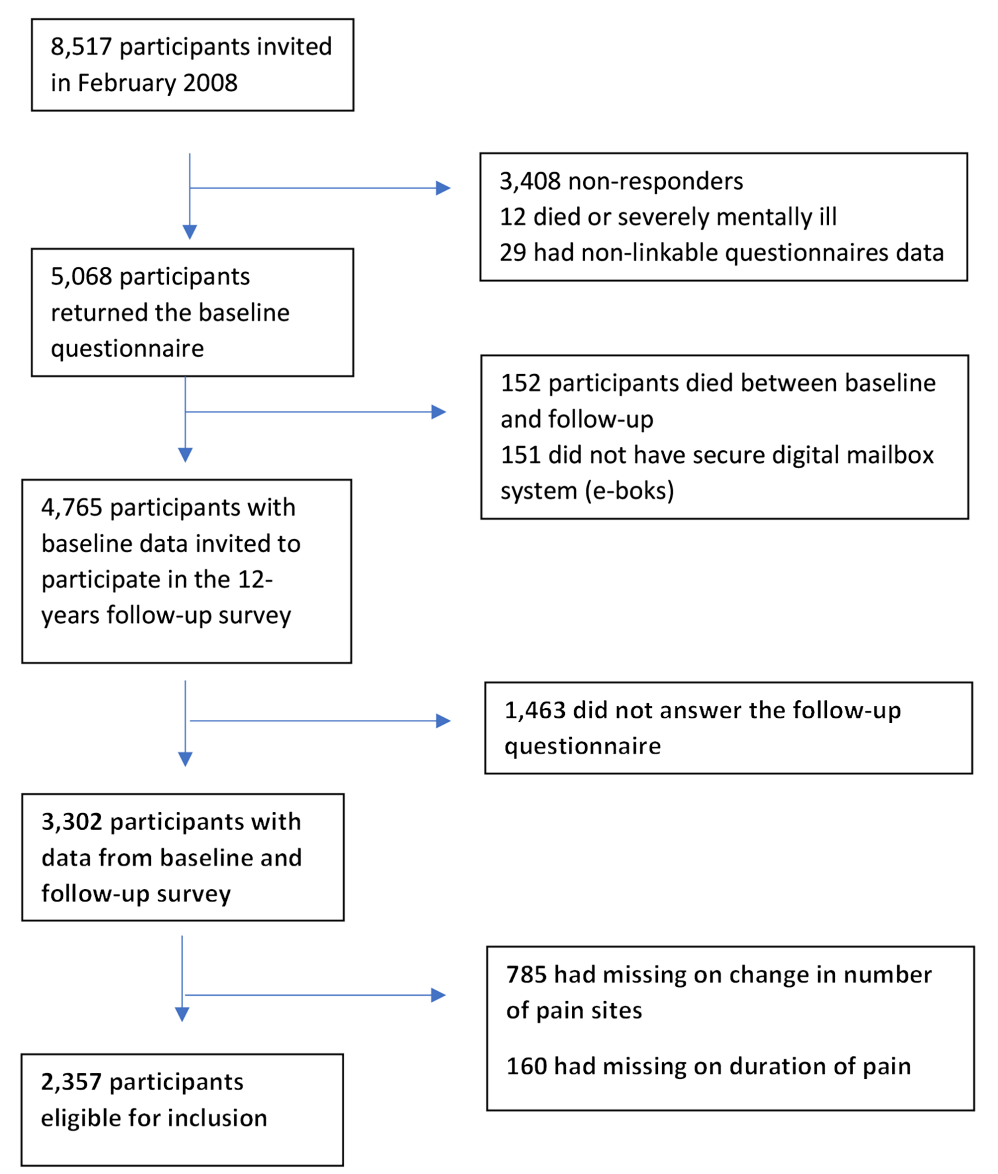
Flowchart of the selection of participants

A total of 3,302 (70.7%) answered the follow-up questionnaire. For a description of non-participants and excluded individuals, please see Fig. 1.

Non-responders to the follow-up questionnaire (*n* = 1,463, 31%) were more often young adults (baseline age < 30 years (22.2% compared to 9.0%) and less often between 40 and 59 years of age at baseline (42.5% compared to 60.2%)) and they had a lower level of education compared to responders (data not shown). A total of 785 (24%) had complete or partial missing values on pain location and/or pain intensity at baseline or follow-up and these individuals were excluded. Also, 160 individuals with missing baseline information on the duration of pain were excluded. These excluded individuals were more often women (61% compared to 55%), above 50 years of age (54% compared to 44%) and with higher level of Self-reported general health (60, (inter-quartile range (IQR) 25–60) compared to 25, (IQR 25–60)) compared to study participants. The missingness of other variables is presented in Table 1.

### Participants

Baseline characteristics of the study population (*n* = 2,357) and stratified by duration of pain are shown in Table 1. A total of 1,585 (67%) reported pain for longer than 3 months, while 772 (33%) reported pain for less than three months. There were slightly more women (54%) than men in this sample. A total of 1,700 (72%) of the participants were above 40 years old. This proportion was higher for people with pain for longer than 3 months (76%) and lower (64%) for participants with pain for less than three months. Upper secondary education or skilled worker was the most frequent level of education (52%). About half were under/normal weight (51%) and the vast majority had no comorbidity (95%). A minor proportion of the participants had a high risk of anxiety (15%) and depression (16%). There were little differences in the distribution of sex, educational level, comorbidity, pain intensity, anxiety, and depression between individuals with pain for longer/less than 3 months in this sample. 11% of this sample reported no pain sites at baseline and 43% reported 3–5 pain sites at baseline. The distribution of the baseline number of pain sites was different for participants with a duration of pain of less/longer than 3 months (*p* < 0.005). For participants with pain for less than 3 months at baseline reported 25% no pain sites and 35% three or more pain sites while 4% of participants with pain for longer than 3 months reported no pain sites and 67% reported three or more pain sites. Median level of pain intensity was 4 (2–5 IQR). Participants with pain for less than 3 months had a medium general health of 25 (25–60 IQR), which was lower than participants with pain for longer than 3 months (median: 60 (25–60 IQR)). Additional baseline information stratified by baseline number of pain sites can be found as supplementary material.

**Table 1:** Baseline characteristics of 2,357 participants stratified by duration of pain

		Total		Pain for less than 3 months		Pain for longer than 3 months
Participants, n (%)		**2,357 (100.0)**		**772 (32.8)**		**1,585 (67.2)**
Sex (Female), n (%)		**1,283 (54.4)**		**402 (52.1)**		**881 (55.6)**
Age, n (%)						
<20 years		**75 (3.2)**		**34 (4.4)**		**41 (2.7)**
20–29 years		**155 (6.6)**		**69 (8.9)**		**86 (5.4)**
30–39 years		**427 (18.1)**		**178 (23.1)**		**249 (15.7)**
40–49 years		**658 (27.9)**		**200 (25.9)**		**458 (28.9)**
50–59 years		**731 (31.0)**		**201 (26.0)**		**530 (33.4)**
≥60 years		**311 (13.2)**		**90 (11.7)**		**221 (13.9)**
Educational level, n (%)						
Bachelor/Master/Doctoral		**741 (31.4)**		**266 (34.5)**		**475 (30.0)**
Upper secondary education or skilled worker		**1,236 (52.4)**		**377 (48.8)**		**859 (54.2)**
Primary and lower secondary education		**380 (16.2)**		**129 (16.7)**		**251 (15.8)**
Body mass Index, n (%)						
Under/normal weight		**1,179 (51.4)**		**406 (54.1)**		**773 (50.1)**
Overweight		**851 (37.1)**		**273 (36.4)**		**578 (37.4)**
Obesity		**265 (11.5)**		**72 (9.5)**		**193 (12.5)**
Comorbidity, n (%)						
**No comorbidity**		**2,242 (95.1)**		**743 (96.2)**		**1,499 (94.6)**
**Comorbidity**		**115 (4.9)**		**29 (3.8)**		**86 (5.4)**
Anxiety score, n (%)						
Low score		**1,980 (84.7)**		**678 (88.4)**		**1,302 (82.9)**
High score		**358 (15.3)**		**89 (11.6)**		**269 (17.1)**
Depression score, n (%)						
Low score		**1,965 (84.1)**		**684 (89.3)**		**1,281 (81.5)**
High score		**372 (15.9)**		**82 (10.7)**		**290 (18.5)**
**Number of pain sites, n (%)**						
**No pain sites**		**261 (11.1)**		**198 (25.6)**		**63 (4.0)**
**1 or 2 pain sites**		**750 (31.8)**		**293 (38.0)**		**457 (28.8)**
**3 to 5 pain sites**		**1,010 (42.9)**		**239 (31.0)**		**771 (48.7)**
**6 or 7**		**336 (14.2)**		**42 (5.4)**		**294 (18.5)**
Pain intensity, Median (IQR)		**4 (2–5)**		**3 (1–4)**		**4 (3–5)**
General Health SF-12, Median (IQR)		**25 (25–60)**		**25 (25–60)**		**60 (25–60)**

The numerical variables with normally distributed data are described by means and standard deviation (sd). Numerical variables with data not normally distributed are described by medians and interquartile range (IQR). Missing values (%): General Health: 35 (1.6), Body mass Index: 62 (2.6), Anxiety score: 19 (0.8), Depression score: 20 (0.8)

### Change in the number of pain sites over 12 years

**Fig. 2:**
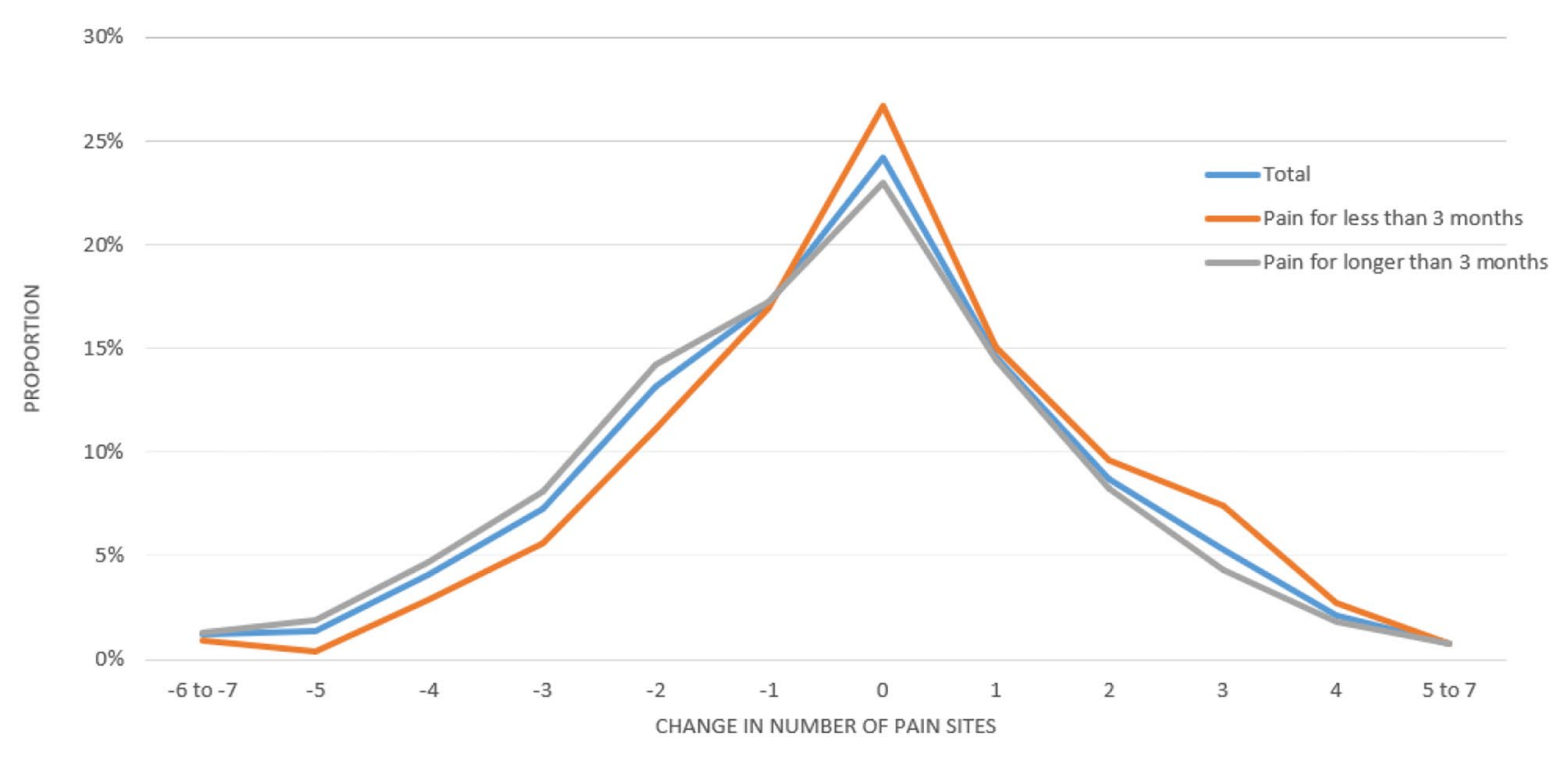
Distribution of change in number of pain sites from 2008 to 2020 The number of pain sites at -7 and − 6 and 5 to 7 are collapsed in this graphical presentation due to the rules of Statistics Denmark not to display data in cells with less than 3 observations

The proportions of change in NPS from 2008 to 2020 in total and stratified by duration of pain can be seen in Fig. 2; Table 2. About 24% of this sample (*n* = 570) had no change in NPS over 12 years, while 56% (*n* = 1,320) reported no change or only an increase/decrease in one pain site over 12 years, and very few (3.4%) reported large changes (5–7 pain sites) over 12 years. Almost half (44%, *n* = 1,044) decreased in NPS over 12 years, while about a third (31.5%, *n* = 743) increased.

We found a mean decrease in NPS of -0.36 (95% CI; -0.44; -0.27) from 2008 to 2020. Participants with pain for less than 3 months had almost no change in NPS (-0.04 (95% CI; -0.18; 0.10)), while participants with pain for longer than 3 months decreased by -0.51 (95% CI; -0.62; -0.41) but the distribution of change in NPS showed the same pattern for both groups (Fig. 2).

**Table 2:** Number of participants with and change in the number of pain sites from 2008 to 2020

Difference between number of pain sites from 2008 and 2020	-6 to -7	-5	-4	-3	-2	-1	0	1	2	3	4	5 to 7	total
Total, n	**28**	**33**	**96**	**171**	**311**	**405**	**570**	**345**	**205**	**125**	**50**	**18**	**2,357**
Pain for less than 3 months, n	**7**	**3**	**22**	**43**	**86**	**131**	**206**	**116**	**74**	**57**	**21**	**6**	**772**
Pain for longer than 3 months, n	**21**	**30**	**74**	**128**	**225**	**274**	**364**	**229**	**131**	**68**	**29**	**12**	**1,585**

### Regression analysis

Table 3 shows the results from the regression analysis. A decrease in NPS over 12 years in the adjusted analysis was statistically significantly associated with pain for longer than 3 months at baseline (-0.27 (95%CI -0.46; -0.09)) and a higher pain intensity at baseline (-0.26 (95%CI -0.32;-0.19)). An increase in NPS over 12 years was statistically significantly associated with age groups between 20 and 49 compared with participants below 20 years of age and this increase was highest for participants between 30 and 39 years of age (0.85 (95% CI: 0.29;1.40)). Also, obesity and general health were statistically significantly associated with an increase in NPS over 12 years. Estimates from all sensitivity analyses were almost identical to Model 2 and analysis with imputed data sets showed similar results as full case analysis (data not shown).

**Table 3:** Linear regression models to predict change in the number of pain sites between 2008 and 2020

Variables ^a^	Model 1: Crude analysis (*n* = 2,357) ^b^	Model 2: Multivariate analysis, all covariates (*n* = 2,244) ^c^
Baseline status for covariates	Coefficient (95% CI)	Coefficient (95% CI)
Duration of pain		
< 3 months	**Ref**	**Ref**
> 3 months	**-0.47 (-0.65; -0.30)**	**-0.27 (-0.46; -0.09)**
Pain intensity	**-0.22 (-0.27; -0.17)**	**-0.26 (-0.32; -0.19)**
Sex (Female)	**-0.10 (-0.26; 0.01)**	**-0.04 (-0.13; 0.22)**
Age		
<20 years	**Ref**	**Ref**
20–29 years	**0.36 (-0.20; 0.93)**	**0.75 (0.16; 1.35)**
30–39 years	**0.59 (0.10; 1.10)**	**0.85 (0.29; 1.40)**
40–49 years	**0.41 (-0.08; 0.90)**	**0.69 (0.15; 1.23)**
50–59 years	**0.10 (-0.39; 0.59)**	**0.33 (-0.21; 0.86)**
≥60 years	**0.14 (-0.38; 0.66)**	**0.30 (-0.26; 0.86)**
Educational level		
Bachelor/Master/Doctoral	**Ref**	**Ref**
Upper secondary education or skilled worker	**-0.16 (-0.34; 0.03)**	**-0.09 (-0.28; 0.10)**
Primary and lower secondary education	**-0.03 (-0.29; 0.22)**	**0.18 (-0.10; 0.45)**
Body mass index		
Under/normal weight	**Ref**	**Ref**
Overweight	**0.07 (-0.12; 0.25)**	**0.13 (-0.05; 0.32)**
Obesity	**0.23 (-0.02; 0.53)**	**0.38 (0.10; 0.65)**
General Health	**-0.004 (-0.008; -0.001)**	**0.005 (0.001; 0.009)**
Comorbidity		
No comorbidity	**Ref**	**Ref**
Comorbidity	**-0.22 (-0.61; 0.17)**	**0.02 (-0.37; 0.42)**
Risk of anxiety		
Low risk	**Ref**	**Ref**
High risk	**-0.41 (-0.64; -0.18)**	**-0.15 (-0.43; 0.13)**
Risk of depression		
Low risk	**Ref**	**Ref**
High risk	**-0.37 (-0.60; -0.15)**	**-0.08 (-0.36;0.19)**

Positive/negative coefficients refers to the increase/reduction in NPS from 2008 to 2020 for each one-unit change in the covariate in question. ^a^ Pain-related factors, demographic, lifestyle, and health-related variables. ^b^ Number of missing values per variable: Body mass Index: 62, General Health: 35, Anxiety score: 19, Depression score: 20. ^c^ The variation in n is due to missing values. Model 2: R^2^: 0.05; Constant: -0.11

## Discussion

This population-based study in adults reporting MSK pain showed that almost 56% of this sample reported no or one change in NPS over 12 years, and very few (3.4%) reported large changes of 5–7 NPS over 12 years. Overall, we found a small mean decrease in NPS in this population over 12 years. We found the duration of pain at baseline to be associated with a change in NPS over this period. However, contrary to our hypothesis, the decrease among participants with pain for longer than 3 months at baseline was higher than for participants with pain of less than 3 months. Adjusted for other included independent variables, the strength of the association between duration of pain and subsequent change in NPS decreased. Participants between 20 and 49 years of age had an increase in NPS over 12 years compared to those aged under 20 years and obesity was associated with an increase in NPS over time compared to under/normal weight. For each incremental increase in pain intensity at baseline, we found a decrease in NPS over the subsequent 12 years and for each incremental increase in general health score at baseline, we found NPS increased over 12 years. Age, obesity, general health, and pain intensity could be candidate prognostic factors for change in NPS.

### Change in the number of pain sites

The results from the present study support previous studies [6, 16–18, 21–25], concluding that individuals with MSK pain tend to experience a persistent number of MSK pain sites across several (1–28) years. Kamaleri et al. [6] found a relatively stable pattern of reporting pain sites across a period of 14 years. They found a small increase in the average number of pain sites between 1990 and 2004, while we found a small decrease. This difference could be explained by the difference in the study population. A recent study by Aili et al. [16] identified trajectories of chronic MSK pain over 21 years and found that most people had stable pain status. Furthermore, the study showed that change in pain status over time is a process and that some with chronic pain improve [16]. This is in line with a study by Landmark et al. [28]. They found that change in chronic multisite pain in most instances is part of an ongoing fluctuation [28]. This could indicate that although the mean change in NPS over time remains relatively stable, NPS for each individual probably fluctuates. We found that about a third (31.5%) experienced an increase in NPS over 12 years, which is in line with findings from others. Christensen et al. [19] found that 29.2% experienced increasing NPS over time. This increase may also reflect fluctuations rather than an actual exacerbation of the pain condition. It could also be that change in NPS over time is influenced by the underlying pain mechanism [48], but such associations have not yet been thoroughly studied.

### Predicting change in the number of pain sites over 12 years

We found that participants with pain for longer than 3 months had a mean decrease in NPS of-0.51 (95% CI; -0.62; -0.41 while participants with pain for less than 3 months had almost no change in NPS (-0.04 (95% CI; -0.18; 0.10)). We consider this to be a relatively small change in both cases. The reason why participants with pain for longer than 3 months had a higher decrease in NPS over time than participants with pain for less than 3 months could be related to the higher NPS at baseline for participants with pain for longer than 3 months and the fact that the measurement of change in NPS introduce both floor and ceiling limits.

In line with previous research, we found age [6, 20, 25, 27, 28, 30, 31], pain intensity [19, 28] and obesity [20, 21, 30, 31] to be predictive of change in NPS over time. Still, there is some inconsistency across prognostic studies. Larsson et al. 2012 [27] conducted a systematic review of risk factors associated with transitioning from regional musculoskeletal pain to chronic multisite pain. They found age, sex, depression, family history of pain and higher number of pain sites at baseline to be prognostic factors for transitioning from chronic regional pain to chronic multisite pain. However, they also found that the number of studies that were unable to confirm female sex and age as prognostic factors exceeded the number of confirmatory studies [27]. We also did not find sex to be a prognostic factor for change in NPS over time and our results also indicate that baseline measurement of number of pain sites is associated with the change in NPS.

The present study is based on a previously established cohort and therefore several interesting variables were not available. This includes lifestyle factors like sleep, smoking and physical activity [17, 21], work-related factors [20, 22, 23], vitality/fatigue [16, 29] and social support [16, 18]. Including these other potential predictors in the adjusted analysis could have changed the results [49].

In contrast to other studies [19, 28], we found that higher pain intensity was associated with a decrease in NPS over 12 years. Landmark et al. [28] found that the risk of developing chronic multisite pain was more than 5 times higher for people reporting moderate or severe pain compared to mild pain, suggesting that moderate or severe pain influences the spread of pain. Our finding could be explained by the ceiling effect on change in NPS because close to 50% of those with pain intensity at 7 (highest possible) had a baseline NPS of 6 or 7, which means they could only remain high or decrease in NPS over time. Christensen et al. [19] found that lower NPS at baseline were more strongly associated with increased NPS over 2 years compared to higher NPS at baseline. Therefore, the operationalization of change in NPS in this study has some inherent limitations and by basing change in NPS on the difference in the reported number of pain sites between baseline (2008) and follow-up (2020), we could have introduced both ceiling and floor problems. A solution to this problem could be to adjust for baseline NPS-score, but by doing so we would most likely induce a spurious correlation between the number of pain sites at baseline and the change in pain sites score, especially in situations where measures of the outcome fluctuate because of measurement error or latent variable instability [50], which is likely to be the case in this study. The results of this study must be interpreted in the light of this issue and our results might differ from studies that have adjusted for baseline NPS-score. Future research should consider alternative scales and methods of analysis to capture the change in the number of pain sites without these limitations.

In the analyses, participants missing answers on pain intensity, duration and location were excluded. The consequence of this was explored by sensitivity analysis where we conducted the same regression analysis on multiple imputation datasets. This analysis showed almost identical estimates. Sensitivity analysis with GH and pain intensity as continuous variables did also not alter any results (data not shown).

Explorative prognostic studies should ideally be based on an inception cohort, where participants are incepted at the onset of an episode of interest, and then followed over time for the development of the outcome [49]. Defining the onset of pain can, however, be a challenge in observational studies. In this study, the measure of baseline NPS was done at the same time as the candidate prognostic factors and for most of the sample pain had started before baseline measurement. However, to explore change in NPS over time in the general population we believe that this sampling approach is reasonable. Evidence also suggests that NPS sometimes fluctuate or recur [28], and we do not know to what degree measurement of NPS at baseline or follow-up represents this fluctuation. It is however likely that regression to the mean will minimize the consequences of this.

### Methodological considerations

The main strength of this study is the large number of participants and a prospective, longitudinal design. The prospective design with 12 years of follow-up ensured that information about the duration of pain and other candidate prognostic factors were collected without knowledge of the outcome, thus differential misclassification is unlikely [51]. Information about age, sex, educational level, and comorbidity was based on registers, ensuring complete data and the use of registers also prevents misclassification since information about exposures is collected independently of information about the outcome.

### Selection problems

The representativity of the sample might have been affected by the response rate since only 59% of the invited participants in 2008 and 69% of the invited participants in 2020 responded to the questionnaire. We found sex, age, and educational level-baseline differences between responders and non-responders. Since women are less likely to recover from chronic pain than men [16], the higher response rate among women might lead to some underestimation of the change in NPS over 12 years. A higher participation rate of people with a higher age and a lower participation rate of people with a lower level of education might also have affected the result of this study. The differences between participants and non-participants in terms of age could have led to an underestimation of change in NPS since higher age is associated with a less increase in NPS compared to younger age [6, 16, 27, 28]. Opposite could difference in level of education have caused an overestimation of change in NPS since a lower level of education is associated with a higher increase in NPS over time compared to higher education [6, 31].

Modest participation rates like the one in this study are not uncommon in large population-based studies and simulation studies suggest that prevalence differences between participants and non-participants do not necessarily have a decisive impact on associations [51, 52]. However, in this case, we cannot rule out the potential for some unquantified selection and the results should be interpreted considering this potential bias. Furthermore, we excluded 945 individuals because of missing values. The excluded group were slightly different than the included participants, however, baseline differences on other descriptive variables between the excluded group and the participants were small. We therefore expect that the potential selection bias due to exclusion is unlikely to have had a decisive impact on the results.

### Informational problems

Some of the variables and the outcome of this study were based on self-reported data, which can lead to information problems [51]. For example, the question about the duration of pain at baseline may have induced some misclassification since the wording is vague. Some of the participants with a duration of pain for longer than 3 months might have been misclassified as having pain for less than 3 months. Furthermore, data in Table 1 support the assumption of some misclassification as 4% of the participants with pain for a longer duration than 3 months but no pain sites and 36% of participants with duration of pain for less than 3 months reported pain in three or more body regions. Another potential information problem is the measurement of comorbidity. The baseline comorbidity index was generated using only 2 years of NPR data even though recommendations for calculating register-based Charlson comorbidity index require 10 years of NPR data [53], however, data 10 years before baseline were not available in this study. This has probably caused an underestimation of comorbidity, but since the information problems of the independent variables in this study are not dependent on the outcome, these potential information problems have probably only led to non-differential misclassification which will most likely cause bias towards the null [51, 54].

### Generalizability and use of results

The Municipality of Odder was in 2008 quite typical for the Danish population [55] and the study population consisted of both adult men and women. The generalizability of the study population to the Danish population is considered to be good. However, the discussed selection problems should be considered when generalizing the results of this study to other populations.

We aimed to identify candidate prognostic factors for change in NPS over time. More knowledge about prognostic factors for change in NPS could help clinicians to identify individuals with a high risk of more rigid patterns of change in NPS and help to guide these patients to management options aiming at managing life in the context of pain.

## Conclusion

In this population-based prospective cohort study in adults, we found that NPS is relatively stable over time as 56% of this sample reported no change or only one pain site increase/decrease over 12 years. Contrary to our hypothesis, we found a mean decrease in NPS over 12 years and this decrease was significant for participants with pain for longer than 3 months. Among other variables pain intensity, age, general health, and obesity could be relevant factors to consider when predicting change in NPS. When interpreting the results of this study, potential selection problems should be considered.

### Electronic supplementary material

Below is the link to the electronic supplementary material.

Supplementary Material 1

## Abbreviations

MSK: Musculoskeletal pain
NPS: Number of pain sites
SEQ: The Standard Evaluation Questionnaire
BMI: Body Mass Index
NPR: The Danish national patient register
CMDQ: The Common Mental Disorder questionnaire
GH: General Health
CPR-number: Danish Civil Personal Registration number
SD: standard deviation
CI: confidence interval
IQR: interquartile range
