# Correction: Change in number of pain sites - which factors are important? A 12-year prospective cohort study

**DOI:** 10.1186/s12891-024-07534-7

**Published:** 2024-06-06

**Authors:** Susanne Vilsbøl, David Høyrup Christiansen, Cecilie Rud Budtz, Johan Hviid Andersen, Søren Mose

**Affiliations:** 1 School of Physiotherapy, VIA University College, Holstebro, Denmark; 2 Centre for Research in Health and Nursing, Research, Regional Hospital Central Jutland, Viborg, Denmark; 3 Department of Clinical Medicine, Health, Aarhus University, Aarhus, Denmark; 4 Elective Surgery Center, Silkeborg Regional Hospital, Silkeborg, Denmark; 5 Department of Occupational Medicine, University Research Clinic, Danish Ramazzini Center, Goedstrup Hospital, Aarhus University, Herning, Denmark

**Correction:** ***BMC Musculoskelet Disord*** **25, 219 (2024)**

10.1186/s12891-024-07344-x

Following the publication of the original article [1], the authors corrected affiliation 3 to remove “Elective Surgery Center, Silkeborg Regional Hospital, Silkeborg, Denmark” as it is a duplicate of affiliation 4. In line with this adjustment to the affiliations, David Høyrup Christiansen should then be affiliated with affiliations 2, 3 and 4.

The correct affiliation 3:

^3^*Department of Clinical Medicine, Health, Aarhus University, Aarhus, Denmark*

The incorrect affiliation 3:

^3^*Department of Clinical Medicine, Health, Aarhus University, Aarhus, Denmark & Elective Surgery Center, Silkeborg Regional Hospital, Silkeborg, Denmark*

The original article [1] has been updated.
