# Supplementary material for: Change in number of pain sites - which factors are important? A 12-year prospective cohort study
Source: BMC Musculoskelet Disord. 2024 Mar 18;25:219. doi: 10.1186/s12891-024-07344-x (PMC10949606; doi:10.1186/s12891-024-07344-x)
Supplement: Supplementary file 1 — Supplementary Material 1 [file 12891_2024_7344_MOESM1_ESM.pdf]

Additional table 1: Baseline characteristics of the study population stratified by number of pain sites at baseline

	Total N=2,302‡	No painsites at baseline N=257	1 or 2 painsites at baseline N=733	3 to 5 painsites at baseline N=984	6 or 7 painsites at baseline N=328	p-value
**Sex, n (%)**						<0.001
**Female**	1,251 (54.3%)	107 (41.6%)	341 (46.5%)	592 (60.2%)	211 (64.3%)	
**Male**	1,051 (45.7%)	150 (58.4%)	392 (53.5%)	392 (39.8%)	117 (35.7%)	
**Age at baseline, n (%)**						0.057
**<20**	73 (3.2%)	10 (3.9%)	19 (2.6%)	32 (3.3%)	12 (3.7%)	
**20-29**	151 (6.6%)	10 (3.9%)	49 (6.7%)	72 (7.3%)	20 (6.1%)	
**30-39**	416 (18.1%)	44 (17.1%)	157 (21.4%)	174 (17.7%)	41 (12.5%)	
**40-49**	649 (28.2%)	66 (25.7%)	210 (28.6%)	280 (28.5%)	93 (28.4%)	
**50-59**	713 (31.0%)	83 (32.3%)	208 (28.4%)	305 (31.0%)	117 (35.7%)	
**>60**	300 (13.0%)	44 (17.1%)	90 (12.3%)	121 (12.3%)	45 (13.7%)	
**Level of Educational, n (%)**						<0.001
**Primary and lower secondary education**	372 (16.2%)	34 (13.2%)	106 (14.5%)	166 (16.9%)	66 (20.1%)	
**Upper secondary education or skilled worker**	1,205 (52.3%)	121 (47.1%)	362 (49.4%)	525 (53.4%)	197 (60.1%)	
**Bachelor/Master/Doctorial**	725 (31.5%)	102 (39.7%)	265 (36.2%)	293 (29.8%)	65 (19.8%)	
**Body Mass Index, n (%)**						0.030
**Under/Normal weight**	1,152 (50.0%)	134 (52.1%)	375 (51.2%)	487 (49.5%)	156 (47.6%)	
**Pre-obesity**	832 (36.1%)	92 (35.8%)	276 (37.7%)	349 (35.5%)	115 (35.1%)	
**Obesity**	260 (11.3%)	26 (10.1%)	61 (8.3%)	120 (12.2%)	53 (16.2%)	
**Missing**	58 (2.5%)	5 (1.9%)	21 (2.9%)	28 (2.8%)	4 (1.2%)	
**Comorbidity, n (%)**						0.14
**No Comorbidity**	2,194 (95.3%)	251 (97.7%)	702 (95.8%)	933 (94.8%)	308 (93.9%)	
**Comorbidity**	108 (4.7%)	6 (2.3%)	31 (4.2%)	51 (5.2%)	20 (6.1%)	
**Level of Anxiety, n (%)**						<0.001
**Low score**	1,948 (84.6%)	252 (98.1%)	678 (92.5%)	815 (82.8%)	203 (61.9%)	
**High score**	354 (15.4%)	5 (1.9%)	55 (7.5%)	169 (17.2%)	125 (38.1%)	
**Level of Depression, n (%)**						<0.001
**Low score**	1,933 (84.0%)	247 (96.1%)	670 (91.4%)	801 (81.4%)	215 (65.5%)	
**High score**	369 (16.0%)	10 (3.9%)	63 (8.6%)	183 (18.6%)	113 (34.5%)	
**Duration of pain, n (%)**						<0.001
**Pain for less than 3 months**	758 (32.9%)	194 (75.5%)	288 (39.3%)	234 (23.8%)	42 (12.8%)	
**Pain for longer than 3 months**	1,544 (67.1%)	63 (24.5%)	445 (60.7%)	750 (76.2%)	286 (87.2%)	
**Pain intensity, Median (IQR)**	4 (2-5)	1 (1-1)	3 (2-4)	4 (3-5)	5 (4-6)	<0.001
**General Health SF-12, Median (IQR)**	25 (25-60)	25 (0-25)	25 (25-60)	60 (25-60)	60 (60-85)	<0.001

‡ Individuals with missing values on anxiety score, depression score and general health (n=55) have been excluded from this table due to the rules of Statistics Denmark not to display data in cells with less than 3 observations. Numerical variables are described by medians and interquartile range (IQR).
